# Associations of Lipoprotein Lipase Gene rs326 with Changes of Lipid Profiles after a High-Carbohydrate and Low-Fat Diet in Healthy Chinese Han Youth 

**DOI:** 10.3390/ijerph110404544

**Published:** 2014-04-23

**Authors:** Xing-chun Zhu, Jia Lin, Qian Wang, Hui Liu, Li Qiu, Ding-zhi Fang

**Affiliations:** 1 Department of Biochemistry and Molecular Biology, West China School of Preclinical and Forensic Medicine, Sichuan University, Chengdu 610041, China; E-Mails: zhuxingch@aliyun.com (X.-C.Z.); jiajiahu198247@163.com (J.L.); wangqianhunya@gmail.com (Q.W.); liuhui1913@126.com (H.L.); qinger636783@sina.com (L.Q.); 2 Clinical Laboratory, Nanchong Central Hospital, Second Affiliated Hospital of North Sichuan Medical College, Nanchong 637000, China

**Keywords:** apolipoproteins, HC/LF diet, high density lipoprotein cholesterol, single nucleotide polymorphism, lipoprotein lipase

## Abstract

To investigate the effects of a high-carbohydrate and low-fat (HC/LF) diet on plasma lipids and apolipoproteins (Apos) of healthy Chinese Han youth with different genotypes of lipoprotein lipase gene (*LPL*) rs326, 56 subjects were given a washout diet of 30.1% fat and 54.1% carbohydrate for seven days, followed by the HC/LF diet of 13.8% fat and 70.1% carbohydrate for six days, with no total energy restriction. Plasma glucose, triglyceride (TG), total cholesterol (TC), high density lipoprotein cholesterol (HDL-C), low density lipoprotein cholesterol (LDL-C), Apo B-100 and Apo A-I were analyzed at baseline and before and after the HC/LF diet. The results show that, when compared with before the HC/LF diet, only the male G carriers experienced increased HDL-C (*p* = 0.008) and Apo A-I (*p* = 0.005) after the HC/LF diet. Decreased TC in both males and females and increased TG in females were found regardless of the genotype after the HC/LF diet. LDL-C decreased in all the subjects although the decrease was not significant in the female G carriers. These results demonstrate that the G allele of *LPL* rs326 associates with the elevated levels of HDL-C and Apo A-I after the HC/LF diet in males of the healthy Chinese Han Youth.

## 1. Introduction

Atherosclerosis (AS) and related vascular diseases are the leading causes of death all over the world [1]. Previous studies have indicated that perturbed concentrations of plasma lipids and apolipoproteins (Apos) are important risk factors for coronary artery disease (CAD) [2,3]. Hypertriglyceridemia (HTG), characterized by elevated plasma levels of triglyceride (TG) and lowered plasma concentrations of high density lipoprotein cholesterol (HDL-C), contributes to the increased risk of CAD [4,5]. It has been reported that HTG can be induced by high-carbohydrate and low-fat (HC/LF) diets [6]. However, evidences showed that although more than 67% of dietary energy was derived from carbohydrate [7], there were lower prevalence and mortality of CAD in Chinese population [8,9]. It is generally believed that these observations are the reflection of their living environments and genetic backgrounds, and suggest the interactions of environmental and genetic factors on modulating lipid metabolism and on development of CAD. 

A key protein involved in the hydrolysis of serum TG packaged in lipoproteins containing Apo B is lipoprotein lipase (LPL) [10]. Serum LPL activity and mass are associated with TG and HDL-C [11,12]. Available data have indicated that the polymorphisms of LPL gene (*LPL*) are related to the disparity of serum lipid profiles and the risk of CAD [13,14,15]. The latest genome-wide association (GWA) study has identified that rs326 in *LPL* intron 8 is strongly associated with serum TG and HDL-C levels in 4,192 Chinese subjects aged from 40 to 80 years old [16]. Another study revealed that rs326 was involved in longitudinal changes of serum TG and HDL-C levels. The minor allele G of rs326 was associated with slowing down a trajectory in both increasing TG and decreasing HDL-C during 20 years of follow-up [14].

Although the risk of CAD has been steadily increasing in younger populations over the past few decades [17], nearly all of the previous studies on carbohydrate-induced HTG have been focused on middle-aged or senior subjects, as CAD is diagnosed primarily after 45 years of age [18]. Much less efforts have been made in studying the effect of interactions of diets and genetic factors on lipid profiles in young healthy subjects. Therefore, in the present study, a washout diet and the HC/LF diet were used to investigate whether interactions of *LPL* rs326 with diets would be associated with the changes of lipid profiles in a healthy young Chinese Han population.

## 2. Subjects and Methods

### 2.1. Ethics Statement

The study protocol was approved by the Human Research Ethics Committee of Sichuan University and conducted with the understanding, and each of the subjects has signed the informed consent.

### 2.2. Subjects

Volunteers were recruited via advertisements seeking healthy young students in West China Medical Center, Sichuan University. A total of 209 university students aged 20–30 years were recruited and 60 of them who met the recruitment criteria described previously [19] entered the study. Briefly, all of them were apparently healthy as indicated by the medical questionnaire, physical examination and laboratory tests. They were not taking any lipid-lowing drugs or hormones, not consuming alcohol, and not smoking. All of them were Chinese Han people and asked to maintain their sleeping and physical activity in a constant manner during the study period. Fifty-six of these (27 males and 29 females) completed the study and the other four participants did not for personal reasons. 

### 2.3. Study Design

Previous studies indicated that serum TG reached a new steady state and remained constant throughout the period of the diet after a 5–7-day HC/LF diet [20,21]. Meanwhile, a short-term dietary intervention can result in a rapid reversal of metabolic and physiological indexes [22]. As a result, we designed a regime of a 7-day washout diet followed by a 6-day HC/LF diet intervention in the current study. 

### 2.4. Diets

Breakfast, lunch and dinner were provided by the Department of Nutrition, West China Hospital, Sichuan University. Each diet was designed to have constant ratios of proteins, fats, and carbohydrates to total energy. As reported before [19], the percentage of proteins, fats and carbohydrates in the washout diet were 15.8%, 30.1% and 54.1% while in the HC/LF diet, the proportions were 16.2%, 13.8% and 70.1%, respectively (Table 1). All the meals were prepared with local foods. The participants were instructed to eat to their satisfaction as usual at each meal and not to have any other food or drink except water. 

**Table 1:** Composition of the washout diet and the HC/LF diet.

Ingredients	Washout diet	HC/LF diet
Protein (% of total energy intake)	15.8 ± 1.8	16.2 ± 1.6
Total fatty acids (% of total energy intake)	30.1 ± 3.6	13.8 ± 1.4
Saturated fatty acids	7.5 ± 0.9	3.6 ± 0.5
Monounsaturated fatty acids	16.1 ± 1.4	7.3 ± 0.8
Polyunsaturated fatty acids	6.4 ± 1.5	2.8 ± 0.3
Carbohydrate (% of total energy intake)	54.1 ± 2.4	70.1 ± 2.8
Polysaccharide	54.1 ± 2.4	65.7 ± 1.3
Monosaccharide and disaccharide	0	4.4 ± 1.3
Cholesterol (mg/d)	422.0 ± 79.1	179.0 ± 41.1
Fiber (g/d)	11.6 ± 2.3	15.4 ± 3.6
Fatty acid composition (% of total fatty acids by energy intake)		
Palmitic fatty acids (16:0)	15.9 ± 4.4	18.9 ± 5.8
Palmitoleic fatty acids (16:1)	2.1 ± 0.7	2.0 ± 0.4
Stearic fatty acids (18:0)	6.9 ± 1.3	7.4 ± 0.9
Oleic fatty acids (18:1)	30.7 ± 6.5	32.1 ± 3.7
Linoleic fatty acids (18:2)	13.2 ± 3.3	17.0 ± 5.1

### 2.5. Blood Collection and Laboratory Analysis

In the mornings of the day the washout diet started (*i.e.*, at baseline), of the day before the HC/LF diet and of the day after the HC/LF diet, 12 hour-fasting venous blood samples were collected between 7:00 and 8:00 a.m., and height, weight, waist circumference, hip circumference, heart rate and blood pressure, including the systolic blood pressure (SBP) and the diastolic blood pressure (DBP) (using a standard mercury sphygmomanometer with 1 of 5 cuff sizes chosen on the basis of the circumference of the participant’s arm), were measured. Plasma TG, TC, HDL-C, LDL-C, apolipoprotein A-I (Apo A-I), apolipoprotein B-100 (Apo B-100) and glucose were analyzed using the routine methods described previously [19]. Average value of three independent measurements for each variable was used for statistical analyses. The inter- and intra-assays coefficients of variation were less than 6%. Body mass index (BMI, = weight (kg)/[height (m)]^2^) and waist-to-hip ratio [WHR, = waist circumference (cm)/hip circumference (cm)] were calculated.

### 2.6. DNA Extraction and Genotyping

Genomic DNA was isolated from white blood cells using a DNAout kit (Tiandz, China). Variants of *LPL* rs326 were identified by polymerase chain reaction (PCR)-restriction fragment length polymorphism (RFLP) analysis, and verified by DNA sequencing. The primer annealing temperatures was 58 °C and primer sequences were as follows: forward primer, 5′-TACACTAGCAAT GTCTAGCTGA-3′; reverse primer, 5′-TCAGCTTTAGCCCAGAATGC-3′. The amplified 488-bp fragments were digested overnight at 37 °C with 1 unit of *Msp*I (Fermentas, Vilnius, Lithuania). The resulting DNA fragments were 171 and 317-bp in length for the GG genotype and an intact 488-bp fragment for the AA genotype.

### 2.7. Statistical Analysis

The subjects with minor allele homozygosity and heterozygosity were pooled and designated as the G allele carriers. The results were expressed as mean ± standard deviation (S.D.) unless stated otherwise. Normality of data was tested using Shapiro-Wilk test. TG values were log-transformed to normalize their distributions and the transformed values were used in all statistical tests. The *χ*^2^ goodness-of-fit test was used to evaluate the Hardy–Weinberg equilibrium. Chi-Square tests were performed to analyze genotype and allele frequencies between the males and the females. Independent-sample *t*-tests were performed to analyze the difference between the subjects with the AA genotype and the G allele carriers, or between the males and females at baseline and before and after the HC/LF diet. Two-tailed paired *t*-tests were performed to analyze the difference between before and after the HC/LF diet. Statistical significance was defined as *p* ≤ 0.05.

## 3. Results and Discussion

### 3.1. Results

#### 3.1.1. Distribution of the Genotypes and Alleles of *LPL* rs326

Table 2 shows the genotype and allele frequencies of *LPL* rs326 in the study population. No deviation from the Hardy-Weinberg Equilibrium was found in the distribution of genotypes (*p* = 0.075). There were no statistical significances in genotype (*p* = 0.171) and allele (*p* = 0.275) frequencies between the males and the females.

**Table 2:** Allele and genotype frequencies of *LPL* rs326.

Parameter	Total	Hardy-Weinberg *p*	Males	Females	p *
Genotype frequency *n* (%)					
AA	34 (60.7)		16 (59.3)	18 (62.1)	
AG	16 (28.6)	0.075	6 (22.2)	10 (34.5)	0.171
GG	6 (10.7)		5 (18.5)	1 (3.4)	
Allele frequency (%)					
A	75.0		70.4	79.3	0.275
G	25.0		29.6	20.7	

Data presented as *n* (%); * Males *vs*. Females by Chi-Square tests.

#### 3.1.2. Baseline Anthropometric and Biochemical Characteristics

Table 3 presents the baseline anthropometric and biochemical characteristics between the subjects with the AA genotype and the G allele carriers of the polymorphism. There were no significant differences of the variables between the subjects with the AA genotype and the G allele carriers in the whole study population.

**Table 3:** Anthropometric and biochemical characteristics of the subjects.

Variables	AA genotype (*n* = 34)	G allele carriers (*n* = 22)
Age (years)	23.29 ± 1.978	22.27 ± 1.279
Females (*n* (%))	18 (52.94)	11 (50.00)
BMI (kg/m^2^)	20.95 ± 2.81	21.17 ± 4.39
WHR (Waist-to-hip ratio)	0.85 ± 0.06	0.87 ± 0.06
Heart rate (bpm)	71.76 ± 8.71	75.45 ± 11.23
Glucose (mg/dL)	73.15 ± 9.02	70.49 ± 10.34
SBP (mmHg)	110.15 ± 13.00	111.36 ± 11.57
DBP (mmHg)	70.15 ± 9.25	74.32 ± 12.28
TC (mg/dL)	151.73 ± 23.98	150.56 ± 31.10
HDL-C (mg/dL)	65.58 ± 12.10	63.59 ± 15.87
LDL-C (mg/dL)	69.76 ± 30.93	70.80 ± 45.13
TG (mg/dL)	71.64 ± 33.52	83.84 ± 55.19
Apo A-I (mg/dL)	207.78 ± 20.42	199.86 ± 27.13
Apo B-100 (mg/dL)	67.28 ± 21.58	68.52 ± 18.77

Data presented as mean ± S.D. or *n* (%); No statistical differences were found in the subjects with the AA genotype and the G allele carriers.

#### 3.1.3. Effects of the HC/LF Diet on Lipid Profiles of the Subjects with Different Genotypes of *LPL* rs326

Table 4 shows the anthropometric variables and lipid profiles of the males and the females with different *LPL* rs326 genotypes at baseline and before and after the HC/LF diet. No significant differences were found between the G allele carriers and the AA homozygotes at baseline, before the HC/LF diet or after the HC/LF diet in males and females separately. However, the males had higher levels of WHR and lower levels of Apo A-I than the females regardless of the genotype constantly at baseline and before and after the HC/LF diet. The male G carriers had lower levels of HDL-C than the female G carriers constantly at baseline and before and after the HC/LF diet, and the males with the AA genotype had a lower level of HDL-C than the females with the same genotype only at baseline. The males with the AA genotype had lower TC and the male G carriers had lower LDL-C than the female counterparts only after the HC/LF diet.

When compared with those before the HC/LF diet, the male G carriers had significantly increased HDL-C and Apo A-I after the HC/LF diet. TC was significantly decreased regardless of the genotype and gender, and TG significantly increased in all the females after the HC/LF diet. Meanwhile, LDL-C was decreased in all the subjects, although the decrease was not statistically significant in the female G carriers. BMI was decreased in the males with the AA genotype, while glucose decreased in the female G carriers after the HC/LF diet. In addition, there were no statistically significant differences in Apo B-100 regardless of gender and the genotype between before and after the HC/LF diet.

### 3.2. Discussion

Although the risk of CAD has been steadily increased in younger populations over the past few decades [17], much less studies have been made in this population. While dietary intervention has become an effective method of primordial prevention on dyslipidemia [23], the HC/LF diet was found to have different effects on plasma lipids in different subjects [19,24]. The mechanism of the discrepancy has not been elucidated yet. In the current study, plasma lipid profiles were investigated in healthy young Chinese Han subjects with different genotypes of *LPL* rs326 at baseline and before and after the HC/LF diet. Since the effect of this polymorphism could be minor and concealed by the confounders, plasma lipids and Apos of the individuals were compared by paired-samples *t*-tests between before and after the HC/LF diet. Conceivably, other genetic and environmental factors affecting lipids metabolism would remain constant for each individual, especially in such a short time of 6 days of the HC/LF diet. Therefore, the differences of lipid profile responses to the HC/LF diet were most likely attributed to the specific genetic background of individuals [24]. The results showed that the G allele of *LPL* rs326 was associated with elevated levels of HDL-C and Apo A-I after the HC/LF diet in the males of healthy Chinese Han Youth. These results suggest that the HC/LF diet interacts with the variants of *LPL*rs326 to influence lipid profiles in the healthy Chinese Han youth.

**Table 4:** Anthropometric parameters, glucose, lipids and apolipoproteins of the male and female subjects with different *LPL* rs326 genotypes at baseline and before and after the HC/LF diet.

Variables	Males		Females	
	AA genotype (*n* = 16)	G allele carriers (*n* = 11)	AA genotype (*n* = 18)	G allele carriers (*n* = 11)
Age (years)	23.50 ± 2.03	22.18 ± 1.60	23.11 ± 1.97	22.36 ± 0.92
BMI (kg/m^2^)				
Baseline	21.44 ± 3.02	22.48 ± 5.47	20.53 ± 2.62	19.85 ± 2.59
Before HC/LF diet	21.29 ± 3.00	22.35 ± 5.47	20.33 ± 2.62	19.82 ± 2.51
After HC/LF diet	21.17 ± 2.95 ^**^	22.23 ± 5.48	20.23 ± 2.67	19.71 ± 2.33
WHR (Waist-to-hip ratio)				
Baseline	0.88 ± 0.05	0.90 ± 0.06	0.82 ± 0.05 ^*^	0.83 ± 0.04 ^*^
Before HC/LF diet	0.89 ± 0.04	0.91 ± 0.05	0.83 ± 0.03 ^*^	0.83 ± 0.06 ^*^
After HC/LF diet	0.90 ± 0.05	0.91 ± 0.05	0.83 ± 0.03 ^*^	0.84 ± 0.04 ^*^
Glucose (mg/dL)				
Baseline	74.64 ± 8.59	68.10 ± 9.90	71.83 ± 9.42	72.89 ± 10.68
Before HC/LF diet	79.64 ± 10.32	83.37 ± 7.17	78.00 ± 10.49	81.35 ± 7.21
After HC/LF diet	78.27 ± 5.28	78.02 ± 8.09	77.46 ± 8.42	77.45 ± 5.07 ^**^
TG (mg/dL)				
Baseline	80.31 ± 38.91	101.14 ± 73.20	64.41 ± 27.32	66.55 ± 19.52
Before HC/LF diet	78.14 ± 27.82	86.42 ± 49.54	67.61 ± 16.61	62.41 ± 15.20
After HC/LF diet	87.31 ± 36.29	88.09 ± 44.03	81.52 ± 25.25 ^**^	75.48 ± 20.12^ **^
TC (mg/dL)				
Baseline	147.08 ± 23.97	145.14 ± 25.93	155.61 ± 23.97	155.48 ± 35.67
Before HC/LF diet	149.04 ± 24.01	145.15 ± 23.61	158.51 ± 29.46	162.38 ± 22.98
After HC/LF diet	116.59 ± 18.02^ ***^	117.76 ± 21.92^ ***^	131.36 ± 19.43 ^*^^,***^	133.38 ± 23.01 ^***^
LDL-C (mg/dL)				
Baseline	70.53 ± 36.53	61.70 ± 52.02	69.11 ± 26.47	79.90 ± 37.26
Before HC/LF diet	63.90 ± 21.65	68.84 ± 24.66	70.11 ± 21.61	71.37 ± 20.67
After HC/LF diet	55.13 ± 11.08 ^**^	54.28 ± 10.76 ^**^	63.10 ± 14.77 ^**^	64.20 ± 10.98 ^*^
HDL-C (mg/dL)				
Baseline	60.33 ± 9.37	53.95 ± 12.89	69.96 ± 12.58 ^*^	73.23 ± 12.59 ^*^
Before HC/LF diet	53.23 ± 11.70	46.96 ± 8.26	60.71 ± 10.70	59.44 ± 9.05 ^*^
After HC/LF diet	56.65 ± 9.70	51.18 ± 10.61 ^**^	63.16 ± 11.08	61.09 ± 6.20 ^*^
Apo A-I (mg/dL)				
Baseline	199.79 ± 23.59	185.00 ± 28.94	214.00 ± 15.53 ^*^	213.36 ± 17.25 ^*^
Before HC/LF diet	173.00 ± 28.46	159.45 ± 23.57	191.61 ± 24.11 ^*^	193.55 ± 20.26 ^*^
After HC/LF diet	173.81 ± 24.06	165.64 ± 28.09 ^**^	195.33 ± 20.09 ^*^	197.91 ± 25.50 ^*^
Apo B-100 (mg/dL)				
Baseline	66.50 ± 24.85	65.00 ± 20.67	67.89 ± 19.39	71.73 ± 17.23
Before HC/LF diet	56.25 ± 18.11	60.55 ± 23.24	58.00 ± 17.02	61.00 ± 16.08
After HC/LF diet	54.75 ± 19.49	60.55 ± 23.98	57.78 ± 15.92	61.64 ± 18.32

Data presented as mean ± S.D.; ^* ^*p* < 0.05, compared with that of the male with same genotype by independent-samples *t*-tests; ^** ^*p* < 0.05, compared with that before the HC/LF diet in the same genotype by paired-samples *t*-tests; ^*** ^*p* < 0.001, compared with that before the HC/LF diet in the same genotype by paired-samples *t*-tests. No significantly differences were found between the G allele carriers and the AA homozygotes at baseline, before the HC/LF diet or after the HC/LF diet.

A number of studies reported discrepant effects of HC/LF diets containing different types of carbohydrates on lipid profiles [6,25]. The traditional Chinese diet mainly consists of white rice, in which most of the carbohydrate is starch [26]. An association was found between diets containing high starch and lower TC and LDL-C in several studies [27,28]. However, the effects of the high-starch diets on HDL-C and TG were inconsistent [27,28,29,30]. In the present study, we presented significantly increased levels of HDL-C and Apo A-I in the males with the G allele of *LPL* rs326 after the HC/LF diet (Table 4). In addition, no parallel changes were found of their confounders including age, BMI, WHR and plasma levels of glucose (Table 4). Therefore, this finding suggests that the G allele of *LPL* rs326 could interact with the HC/LF diet to elevate the level of HDL-C in healthy Chinese males in their early 20s. Since this polymorphism is in the intron 8 of *LPL*, it is very unlikely that this is a functional mutation [16]. One possibility is that this polymorphism is in linkage disequilibrium with some other functional mutations. For example, *LPL* rs326 was found in linkage disequilibrium with *LPL* rs328 in European population. Similarly, we also found the linkage disequilibrium (D’ = 0.990, r^2^ = 0.414) between rs326 and rs328 in 724 Chinese Han subjects in a cross-sectional study (data not shown). The rs328 mutation regarded as a gain-of-function mutation is associated with the anti-atherogenic lipid profiles [31]. In addition, the elevated HDL-C level after the HC/LF diet might also be due to the adaptation to long-term effects of high carbohydrate diets in this population. It is well known that people in China generally have high dietary carbohydrate intake [6,7] and more favorable lipid profiles, including a higher HDL-C level [32,33]. Taken together, young Chinese males with *LPL* rs326 G allele might be more adaptable to the increase of dietary carbohydrate than those with the AA genotype.

Another important finding of the present study was that when compared with that before the HC/LF diet, there was a significantly increased level of plasma TG in the females after the HC/LF diet, which might be attributable to their higher levels of estrogen. It is indicated that HC/LF diets can adversely increase the plasma concentrations of TG [6] which are mainly hydrolyzed by the key enzyme LPL. However, high levels of estrogen in females significantly inhibit the activity of LPL and retard the hydrolysis of TG by LPL [34].

In the present study, all subjects experienced statistically significantly decreased levels of TC after the HC/LF diet compared to before the HC/LF diet. For LDL-C, there was a statistically significant reduction after the HC/LF diet in the subjects, except for the female G carriers. However, a trend toward reduced level (from 71.37 mg/dL to 64.20 mg/dL) was also observed in the female G carriers. Furthermore, *LPL* is located at chromosome 8p22 [35], a region associated with serum TG and HDL-C levels (http://omim.org/entry/609708). Jackson *et al*. [36] reported that plasma postheparin LPL activity was associated with changes in serum lipid concentrations, especially TG and HDL-C. All these imply that *LPL* rs326 might not be directly associated to the reduced levels of TC and LDL-C induced by the HC/LF diet.

## 4. Conclusions

In summary, our results suggest that the G allele of *LPL* rs326 be associated with increased levels of HDL-C and Apo A-I after the HC/LF diet in the healthy young Chinese Han subjects. Although the analyses were based on a small sample size and thus suggestive, the findings justify the need for future studies with larger sample sizes, which will pave the way to personalized dietary intervention to reduce risks of AS and related vascular diseases in China, a country with a quarter of the worlds’ population.
